# Impact of Molecular Testing on Surgical Decision-Making in Indeterminate Thyroid Nodules: A Systematic Review and Meta-Analysis of Recent Advancements

**DOI:** 10.3390/cancers17071156

**Published:** 2025-03-29

**Authors:** Raisa Chowdhury, Jessica Hier, Kayla E. Payne, Mawaddah Abdulhaleem, Orr Dimitstein, Netanel Eisenbach, Véronique-Isabelle Forest, Richard J. Payne

**Affiliations:** 1 Faculty of Medicine and Health Sciences, McGill University, Montreal, QC H3A 0G4, Canada; 2 Department of Otolaryngology-Head and Neck Surgery, McGill University Health Center, Montreal, QC H4A 0B1, Canada; 3 Department of Otolaryngology-Head and Neck Surgery, Jewish General Hospital, McGill University, Montreal, QC H3T 1E2, Canada; 4 Faculty of Arts, McGill University, Montreal, QC H3A 0G4, Canada; 5 Department of Otolaryngology Head and Neck Surgery, Thyroid and Parathyroid Surgery Division, Dr. Suliman Alhabib Hospital, Jeddah 22245, Saudi Arabia

**Keywords:** thyroid nodule, molecular testing, indetermiante cytology, surgical decision-making, thyroseq, afirma, Thygen X/ThyraMIR

## Abstract

**Background**: The management of indeterminate thyroid nodules (Bethesda III/IV) has evolved with molecular testing, aiming to reduce unnecessary surgeries. However, the comparative effectiveness of different platforms in influencing surgical decision-making remains unclear. This systematic review and meta-analysis evaluate the impact of molecular testing on surgical avoidance rates. **Methods**: A systematic literature search was conducted across eight electronic databases, including Embase, PubMed, and Cochrane Library, from January 2019 to December 2024, following PRISMA guidelines to encompass most recent advancements in the last 5 years. Studies evaluating Afirma Gene Expression Classifier (GEC), Afirma Genomic Sequencing Classifier (GSC), ThyroSeq V2, ThyroSeq V3, and ThyGenX/ThyraMIR were included. The primary outcome was surgical avoidance, analyzed using a random-effects model. **Results**: Thirty-one studies comprising 4464 indeterminate thyroid nodules met inclusion criteria. Pooled surgical avoidance rates varied across platforms: ThyroSeq V2 (50.3%, 95% CI: 20.8–79.6%), ThyroSeq V3 (62.5%, 95% CI: 54.8–70.0%), Afirma GEC (58.8%, 95% CI: 43.6–73.1%), Afirma GSC (50.6%, 95% CI: 34.3–66.8%), and ThyGenX/ThyraMIR (68.6%, 95% CI: 63.1–73.9%). ThyGenX/ThyraMIR had the highest surgical avoidance rate and lowest heterogeneity (I^2^ = 51.2%), while ThyroSeq showed improvement from V2 to V3. **Conclusions**: Molecular testing reduces unnecessary thyroid surgeries, with avoidance rates ranging from 50.3% to 68.6%. While ThyGenX/ThyraMIR showed the highest avoidance rate, its limited representation warrants cautious interpretation. Standardized protocols are needed to optimize clinical application. Further prospective studies should compare platforms and assess long-term outcomes and cost-effectiveness.

## Simple Summary

Thyroid nodules are common, and when their diagnosis is unclear, doctors often recommend surgery to rule out cancer. However, many of these surgeries turn out to be unnecessary, leading to potential complications and increased healthcare costs. Molecular testing has emerged as a promising tool to improve decision-making by predicting whether a nodule is likely to be benign or malignant. Several molecular tests exist, but their effectiveness varies, and it is unclear which test performs best in avoiding unnecessary surgery. This study systematically reviews and analyzes data from 31 studies to compare the ability of different molecular testing platforms to reduce surgery rates. The findings suggest that all molecular tests help avoid surgery in some cases, but their performance differs. While one test (ThyGenX/ThyraMIR) showed the highest surgical avoidance rate, its results should be interpreted cautiously due to limited sample size. More research is needed to optimize the use of molecular testing in clinical practice.

## 1. Introduction

Over the last decade, the clinical management of indeterminate thyroid nodules has undergone significant transformation with the emergence of molecular testing platforms [1,2]. These nodules, classified as Bethesda III (Atypia of Undetermined Significance/Follicular Lesion of Undetermined Significance) or Bethesda IV (Follicular Neoplasm/Suspicious for Follicular Neoplasm) [3], represent a substantial diagnostic challenge, creating uncertainty in surgical decision-making [4]. Historically, this diagnostic uncertainty has led to diagnostic thyroid surgery for most patients with indeterminate cytology, resulting in numerous potentially unnecessary operations and associated healthcare costs [5].

Until recently, the standard approach for patients presenting with indeterminate thyroid nodules was diagnostic surgery, achieving definitive histological diagnosis but potentially subjecting patients to unnecessary surgical intervention and associated risks [6]. However, recognizing the need for more precise diagnostic tools, various molecular testing platforms have emerged, employing approaches ranging from gene expression analysis to next-generation sequencing. This evolution is exemplified by platforms such as Afirma GEC that uses gene expression analysis to classify nodules, while the Afirma GSC incorporates genomic sequencing for a more detailed genetic assessment. ThyroSeq V2 and V3 utilize next-generation sequencing to identify a broad spectrum of genetic alterations [7]. ThyGenX/ThyraMIR combines genetic analysis with microRNA profiling, offering a comprehensive approach to risk stratification [7].

These molecular platforms aim to improve preoperative risk stratification and reduce unnecessary surgeries through distinct methodologies to enhance diagnostic accuracy and guide surgical decision-making [8]. However, despite their growing implementation, comprehensive evidence regarding molecular testing’s effect on surgical avoidance rates remains limited [9].

Recognizing that the appropriate utilization of molecular testing remains a subject of debate, we aim to systematically review the literature evaluating the impact of molecular testing on surgical decision-making for patients with indeterminate thyroid nodules. Specifically, this review seeks to assess surgical avoidance rates following molecular testing and determine whether implementation of these platforms can effectively reduce unnecessary surgical interventions. This knowledge is crucial for institutions making informed decisions about platform selection and implementation strategies in clinical practice. Our study aligns with the 2015 ATA guidelines and ETA recommendations which advocate for molecular testing in Bethseda III/IV nodules to guide surgical decision-making [9]. Consistent with these guidelines, we prioritized platforms validated in large multicenter trials, like Thyroseq V3 and Afirma GSC, and excluded studies lacking histopathological confirmation. Previous reviews have focused primarily on individual platforms or earlier versions, leaving a critical gap in understanding the comparative effectiveness of current molecular testing platforms

## 2. Materials and Methods

### 2.1. Literature Search

The systematic review of the literature was performed according to the Preferred Reporting Items for Systematic Reviews and Meta-Analyses (PRISMA) guidelines [10]. The protocol was registered in PROSPERO under registration number CRD42024615384. The search was conducted across eight electronic databases (Embase, PubMed, Web of Science, Crossref, CINAHL, Cochrane Library, Scopus, and ClinicalTrials.gov) from January 2019 to December 2024. The selected timeframe was chosen to reflect most recent advancements in molecular testing technologies. The search strategy utilized keywords and Medical Subject Headings (MeSH) terms related to three main categories: population terms (“thyroid nodule*”, “indeterminate thyroid*”, “Bethesda III”, “Bethesda IV”, “AUS/FLUS”, “FN/SFN”), intervention terms (“molecular test*”, “molecular marker*”, “Afirma”, “ThyroSeq”, “ThyGenX”, “ThyraMIR”), and outcome terms (“surgery”, “surgical decision*”, “surgical management”, “surgical avoid*”). The search was supplemented by manual screening of reference lists from included articles and recent reviews to ensure comprehensive coverage.

### 2.2. Screening and Eligibility Assessment

Studies were included if they met the following criteria: (i) published between 2019 and 2024; (ii) evaluated molecular testing platforms (Afirma GEC, Afirma GSC, ThyroSeq V2, ThyroSeq V3 or ThyGenX/ThyraMIR) in indeterminate thyroid nodules; (iii) reported surgical avoidance rates directly or provided sufficient data to calculate these rates from surgical resection data; (iv) included adult patients with Bethesda III (AUS/FLUS) or IV (FN/SFN) cytology; and (v) included a minimum follow-up period of 6 months for non-operative management.

Studies were excluded if they: (i) were case reports, reviews, editorials, or conference abstracts; (ii) included pediatric populations; (iii) evaluated molecular testing platforms other than the five specified platforms (except for two studies using custom NGS panels); (iv) lacked surgical decision-making outcomes or sufficient data to calculate surgical avoidance rates; (v) had overlapping patient populations with other included studies; or (vi) were published in languages other than English. Additionally, studies with inadequate data reporting for meta-analysis were excluded.

### 2.3. Quality Assessment and Risk of Bias

Risk of bias assessment was conducted independently by two reviewers using the Newcastle–Ottawa Scale [11] for observational studies and the Cochrane Risk of Bias tool for randomized controlled trials [12]. Publication bias was assessed using funnel plots and Egger’s test [13] for each molecular testing platform.

### 2.4. Data Extraction

Data extraction was facilitated using Covidence, a systematic review management software [14]. Two independent reviewers (R.C. and M.A.) performed initial screening of titles and abstracts, followed by full-text review of potentially eligible studies. Data were extracted into standardized forms, capturing study characteristics, patient demographics, nodule characteristics, molecular testing platform used, and surgical outcomes. The primary outcome measure was surgical avoidance rate. Authors were contacted when relevant data were unclear or missing.

### 2.5. Statistical Analysis

Statistical analysis employed random-effects model using JBI SUMARI (accessed February 2025) and Comprehensive Meta-Analysis software version 4.0 [15,16,17]. Effect measures were pooled using the DerSimonian and Laird method, with surgical avoidance rates calculated for each platform and presented with 95% confidence intervals. Heterogeneity was assessed using the I^2^ statistic, with values of 25%, 50%, and 75% considered as low, moderate, and high heterogeneity, respectively [18].

### 2.6. Molecular Testing Platforms

To account for methodological differences between platforms, we extracted detailed information about each test from the included studies and relevant publications. The key characteristics of each platform are summarized below.

Afirma GEC: This platform analyzes the expression levels of 167 genes using microarray technology. The test classifies nodules as either benign or suspicious based on the proprietary Gene Expression Classifier (GEC) algorithm [6,7].

Afirma GSC: This platform uses next-generation sequencing to analyze RNA from 555 genes. The test detects gene mutations, fusions, and expression alterations. Results are classified as benign, suspicious, or non-diagnostic using the Genomic Sequencing Classifier (GSC) algorithm [6,7].

Thyroseq V2 and V3: These platforms employ next-generation sequencing to detect DNA and RNA alterations in a panel of genes commonly implicated in thyroid cancer. ThyroSeq V3 has an expanded gene panel compared to V2. Both versions report the presence or absence of specific mutations, gene fusions, and gene expression alterations [5,6,7].

ThyGenX/ThyraMIR: This platform combines mutation analysis using next-generation sequencing with microRNA expression profiling. The test analyzes mutations in key thyroid cancer genes (including *BRAF*, *RAS*, *TERT*, *TP53*, and *PIK3CA*) and the expression levels of 10 microRNAs (including miR-29b, miR-146b, miR-221, and miR-222). Results are integrated using a proprietary algorithm to provide a risk score for malignancy [5,6,7].

## 3. Results

### 3.1. Summary of Literature Search

**Figure 1:**
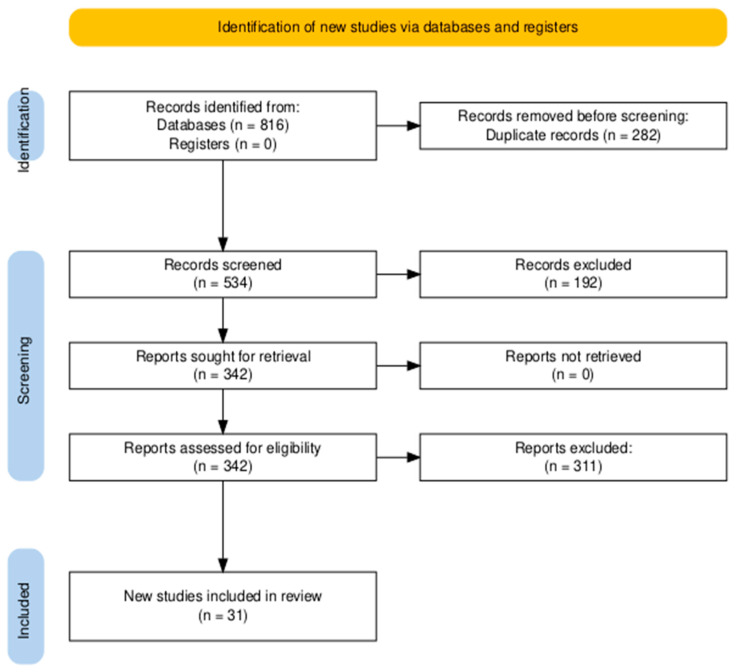
The Preferred Reporting Items for Systematic Reviews and Meta-Analyses (PRISMA) flowchart for literature search and study selection.

The systemic database search yielded 816 records from databases, with no additional records identified through registers. After removing 282 duplicate records, 534 articles were screened based on titles and abstracts. Of these, 342 full-text articles were assessed for eligibility. Following detailed evaluation, 311 articles were excluded based on predefined criteria. Ultimately, 31 studies [19,20,21,22,23,24,25,26,27,28,29,30,31,32,33,34,35,36,37,38,39,40,41,42,43,44,45,46,47,48,49] met the inclusion criteria and were included in the final review (Figure 1).

### 3.2. Study Characteristics

The systematic review encompassed 31 studies published between 2019 and 2024, with the following distribution: 2 experimental studies (1 randomized clinical trial, 1 secondary RCT analysis) and 29 observational studies (2 prospective cohort studies, 25 retrospective cohort studies, 2 multi-institutional retrospective analyses). The study populations demonstrated median ages ranging from 47.8 to 59.3 years with 63% to 86% female predominance and nodule sizes ranging from 1.8 to 2.7 cm, with sample sizes varying from 27 to 1593 nodules. Studies were primarily conducted at North American academic medical centers and specialized endocrine surgery units, with additional studies from China, India, and Chile, spanning durations of 12 to 84 months between 2015 and 2022, with follow up periods of 6 to 72 months and majority reporting minimum 12 month follow up. The distribution of indeterminate nodules followed Bethesda classification, with Bethesda III (AUS/FLUS) comprising 46% to 85% of cases, Bethesda IV (FN/SFN) representing 15% to 42%, and Hürthle cell neoplasms accounting for 6% to 16% when specifically reported. The review evaluated five primary molecular testing platforms: Afirma Gene Expression Classifier (GEC) in 6 studies, Afirma Genomic Sequencing Classifier (GSC) in 6 studies, ThyroSeq V2 in 3 studies, ThyroSeq V3 in 10 studies, ThyGenX/ThyGeNEXT ThyraMIR in 4 studies, and other platforms (including custom NGS panels) in 2 studies (Table 1).

### 3.3. Summary of Quality Assessment

The quality assessment of included studies revealed consistent patterns across different study types. For the 29 observational studies evaluated using the Newcastle–Ottawa Scale (NOS), most studies demonstrated adequate representativeness and selection of study groups, scoring 3–4 stars in the selection domain. In the comparability domain, studies generally scored 1–2 stars, with variation observed in how well they controlled for confounding factors. For outcome assessment, studies typically scored 2–3 stars, showing adequate follow-up periods but some limitations in outcome validation. The two experimental studies assessed using the Cochrane Risk of Bias tool showed low risk for random sequence generation in both studies, with allocation concealment being low risk in one study and unclear in the other. Overall, 25 out of 31 studies were of moderate to high quality, though common limitations included lack of validation in outcome measurement, incomplete test–retest reliability assessment, and variable approaches to controlling for confounding factors. The assessment revealed that while most studies maintained acceptable methodological standards, there were consistent limitations in outcome validation and reliability testing.

### 3.4. Surgical Avoidance Rates

#### 3.4.1. Throseq V2

**Figure 2:**
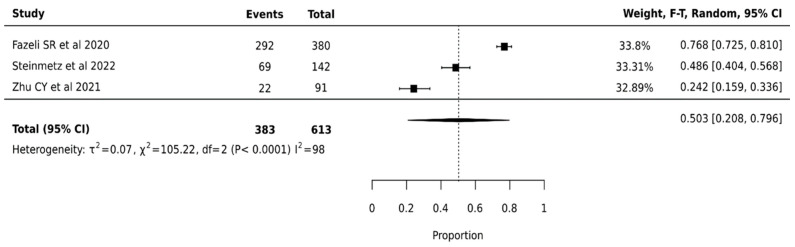
Thyroseq V2—Forest Plot [26,44,49].

ThyroSeq V2 demonstrated a pooled surgical avoidance rate of 50.3% (95% CI: 20.8–79.6%) across 613 nodules from three studies. The wide confidence interval suggests considerable uncertainty in the true effect size. Statistical analysis revealed substantial heterogeneity (I^2^ = 98%, *p* < 0.0001), indicating significant variation in platform performance across institutions (Figure 2).

#### 3.4.2. Thyroseq V3

**Figure 3:**
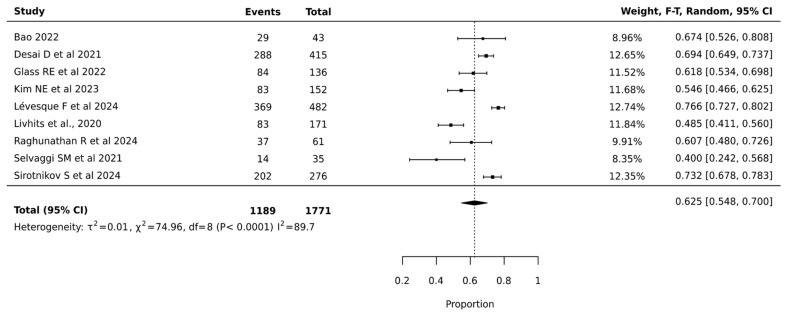
Thyroseq V3—Forest Plot [21,25,27,29,30,32,39,41,42].

Meta-analysis of 1771 nodules across nine studies showed that ThyroSeq V3 achieved a pooled surgical avoidance rate of 62.5% (95% CI: 54.8–70.0%). The narrower confidence interval compared to V2 suggests more precise estimation. Heterogeneity remained high but improved (I^2^ = 89.7%, *p* < 0.0001, τ^2^ = 0.01), indicating more consistent performance across institutions. The substantial statistical heterogeneity indicated significant variation in ThyroSeq V3’s effectiveness across different clinical settings, suggesting that institutional expertise and patient selection critically influenced surgical decision-making outcomes. The relatively narrow confidence interval of the pooled estimate supports the reliability of ThyroSeq V3 in reducing unnecessary surgeries for indeterminate thyroid nodules (Figure 3).

#### 3.4.3. Afirma GEC

**Figure 4:**
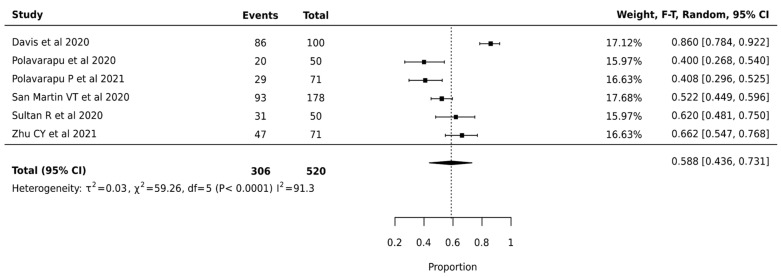
Afirma GEC—Forest Plot [24,37,38,40,45,49].

Meta-analysis of 520 nodules from 6 studies revealed a pooled surgical avoidance rate of 58.8% (95% CI: 43.6–73.1%). High heterogeneity was observed (I^2^ = 91.3%, *p* < 0.0001, τ^2^ = 0.03), suggesting variable effectiveness across different clinical settings (Figure 4).

#### 3.4.4. Afirma GSC

**Figure 5:**
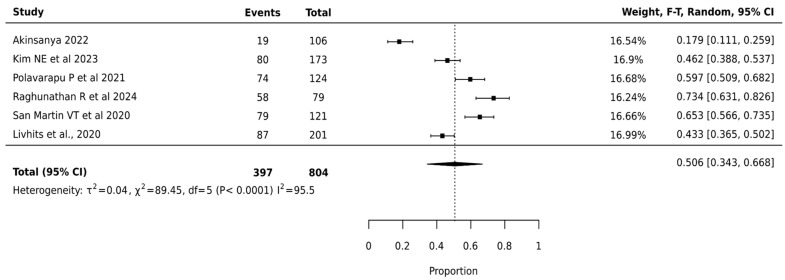
Afirma GSC—Forest Plot [20,29,32,38,39,40].

The meta-analysis of Afirma GSC involving 804 nodules across 6 studies showed a pooled surgical avoidance rate of 50.6% (95% CI: 34.3–66.8%). The platform demonstrated the highest heterogeneity among all platforms (I^2^ = 95.5%, *p* < 0.0001), indicating significant variation in institutional performance (Figure 5).

#### 3.4.5. ThyGenX/ThyraMIR

**Figure 6:**
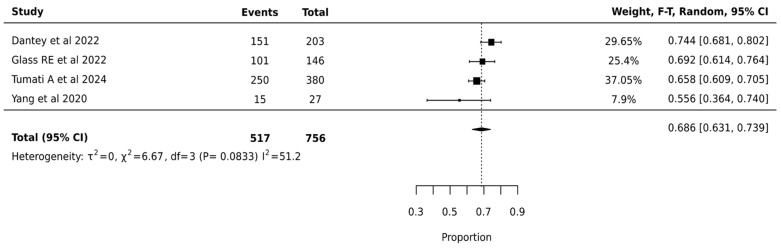
ThyGenX/ThyraMIR—Forest Plot [23,27,47,48].

ThyGenX/ThyraMIR showed the highest pooled surgical avoidance rates of 68.6% (95% CI: 63.1–73.9%) after analysis of 756 nodules from 4 studies. The moderate statistical heterogeneity (I^2^ = 51.2%, *p* = 0.0833) suggested some variation in ThyGenX/ThyraMIR’s effectiveness across different clinical settings, though less pronounced than other molecular testing platforms. The relatively narrow confidence interval of the pooled estimate (63.1–73.9%) and lower heterogeneity compared to other platforms indicate more consistent performance of ThyGenX/ThyraMIR in surgical decision-making for indeterminate thyroid nodules (Figure 6).

### 3.5. Mutation Frequencies Across Platforms and Coherence with Bethseda Classifications

The pooled analysis of BRAF and RAS mutations across different platforms revealed significant variability in mutation frequencies, highlighting the influence of platform-specific detection capabilities and study designs. For BRAF mutations, Thyroseq demonstrated a pooled mutation proportion of 2.7% (95% CI: 0.3%, 6.7%), with high heterogeneity (I^2^ = 85.5%), while NGS showed a higher pooled mutation proportion of 6.7% (95% CI: 3.4%, 10.7%), with no heterogeneity (I^2^ = 0%). For RAS mutations, ThyroSeq showed a pooled mutation proportion of 18.8% (95% CI: 9.3%, 30.6%), with significant heterogeneity (I^2^ = 95.7%), whereas NGS demonstrated a proportion of 15.7% (95% CI: 3.8%, 32.9%), with moderate heterogeneity (I^2^ = 67.1%). However, due to the lack of detailed data on mutation frequencies stratified by Bethesda categories in the included studies, the coherence of specific gene mutations with Bethesda classifications could not be fully addressed, limiting the ability to draw definitive conclusions about their diagnostic or prognostic implications (Table 2).

## 4. Discussion

This meta-analysis evaluated the impact of molecular testing on surgical decision-making in indeterminate thyroid nodules. The analysis included 31 studies and 4464 indeterminate thyroid nodules, assessing the impact of molecular testing on surgical decision-making. The analysis revealed that ThyGenX/ThyraMIR had the highest surgical avoidance rate at 68.6% (95% CI: 63.1–73.9%), with the lowest heterogeneity (I^2^ = 51.2%). This superior performance likely stems from its combined approach, utilizing both mutation analysis and microRNA expression [50]. However, the sample size for ThyGenX/ThyraMIR was smaller compared to ThyroSeq and Afirma, which may limit the generalizability of its results.

ThyroSeq showed improvement from V2 to V3, with rates increasing from 50.3% (95% CI: 20.8–79.6%) to 62.5% (95% CI: 54.8–70.0%). This advancement reflects enhanced genomic sequencing capabilities and refined algorithmic interpretation.

Afirma platforms showed comparable performance between iterations, with GEC achieving 58.8% (95% CI: 43.6–73.1%) and GSC showing 50.6% (95% CI: 34.3–66.8%). Significant heterogeneity [18] observed across most platforms (I^2^ ranging from 51.2% to 98%) indicates substantial variation in real-world effectiveness. This variability likely stems from differences in institutional expertise, patient selection criteria, and implementation protocols. The notably lower heterogeneity in ThyGenX/ThyraMIR suggests more consistent performance across different clinical settings, suggesting variation in implementation effectiveness. The risk of bias was assessed using the NOS and Cochrane Risk of Bias tool, with most studies being observational and of moderate to high quality. A few studies reported complication rates, but these did not show significant differences.

Our findings demonstrating the surgical avoidance rate observed for ThyroSeq V3 (62.5%) aligned with a similar study by Chen et al. (2020) [51], which showed ThroSeq V3’s contribution in preventing unnecessary diagnostic surgeries by classifying indeterminate thyroid nodules as either negative or positive. In addition, our study aligned with a meta-analysis by Vardarli et al. (2024) [52] that showed that molecular testing in patients with indeterminate thyroid nodules helps avoid unnecessary thyroid surgery, with Thyroseq V3 having the best diagnostic performance. The findings that ThyGen/ThyraMIR had the highest surgical avoidance rate in comparison with other molecular tests, corresponded with findings by Glass et al. (2021) [27] who reported that ThyGenX/ThyraMIR had a surgical avoidance rate of 38.9% compared with Thyroseq at 24.2%. 

This analysis underscores the potential of molecular testing to substantially reduce unnecessary thyroid surgeries [52], with observed surgical avoidance rates ranging from 50.3% to 68.6% across different platforms. The consistent performance of ThyGenX/ThyraMIR, with the highest surgical avoidance rate (68.6%), suggests that this platform can offer more reliable clinical decision-making support, aiding in surgical planning and patient counseling. However, given the relatively limited number of studies assessing this platform, further validation in larger, multi-center cohorts is necessary before drawing definitive conclusions. The demonstrated improvement from ThyroSeq V2 to V3 further highlights the clinical value of platform evolution in enhancing diagnostic precision. While these findings support the adoption of molecular testing, the observed variation in surgical avoidance rates across institutions emphasizes the need for standardized implementation protocols and integration of test results within a multidisciplinary team, ultimately ensuring optimal patient selection and surgical outcomes [53]. Moreover, providing patients with clear and comprehensive information about their treatment options and the role of molecular testing is crucial for facilitating informed decision-making and promoting active coping strategies, which can reduce anxiety and improve patient satisfaction [54,55].

Our meta-analysis is strengthened by its comprehensive search strategy across multiple databases and rigorous data extraction and quality assessment. However, the predominance of observational studies (29 out of 31) limits causal inference and introduces potential selection bias. Sample sizes varied significantly across studies and platforms, with limited representation for ThyroSeq V2 and ThyGenX/ThyraMIR, making direct comparisons less robust. The substantial variation in follow-up periods (6 to 84 months) also restricts the assessment of long-term outcomes. While publication bias was minimal, the substantial heterogeneity observed in most platforms, coupled with the geographic concentration of studies in North America, suggests that unmeasured factors and limited generalizability may influence the findings [56]. The lack of standardized reporting further complicates the interpretation and application of results across different clinical settings. While we attempted to analyze the impact of specific mutations on surgical avoidance rates, the available data were limited. Our analysis of *BRAF* and *RAS* mutation frequencies revealed variations across platforms. Due to inconsistencies in reporting and a lack of stratification by Bethesda category, we were unable to draw definitive conclusions about the relationship between specific mutations, platform performance, and surgical outcomes. With molecular testing significantly reducing unnecessary surgeries, there remains a risk of false-negative results, where a malignant nodule is incorrectly classified as benign. The accuracy of each molecular testing platform, including its ability to correctly identify benign nodules and avoid unnecessary surgeries, is crucial. ThyGenX/ThyraMIR demonstrated a high surgical avoidance rate; however, to mitigate the risk of missing malignant nodules, clinical practice should integrate molecular testing with thorough clinical and radiological evaluations. Regular follow-up is also essential for nodules.

Future research should focus on standardizing implementation protocols for molecular testing and conducting long-term outcome assessments to evaluate the durability of surgical avoidance. Prospective studies comparing different molecular testing platforms head-to-head are needed to provide more definitive evidence on their relative effectiveness. Furthermore, research should investigate the cost-effectiveness of molecular testing strategies and explore the impact of molecular testing on patient-reported outcomes and quality of life.

## 5. Conclusions

This systematic review and meta-analysis support the role of molecular testing as a valuable adjunct in the management of indeterminate thyroid nodules, contributing to improved risk stratification and reduction in unnecessary surgeries when interpreted alongside established clinical guidelines. While surgical decisions remain closely guided by the ATA and ETA frameworks, the integration of molecular testing—particularly platforms such as ThyroSeq V3 and ThyGenX/ThyraMIR—has demonstrated the potential to influence clinical management, especially in the presence of specific mutations such as BRAF. However, the broader use of multigene panels has yet to consistently impact decision-making independently.

Our findings highlight the heterogeneity in test performance and institutional practices, underscoring the need for standardized implementation protocols and clearer clinical algorithms. Looking ahead, the incorporation of additional mutations such as TERT and expanded genomic classifiers may enhance the predictive value of these platforms. Until such tools are validated through prospective studies, molecular testing should be applied as a complementary measure within multidisciplinary evaluation frameworks, ensuring informed and individualized patient care.

## Figures and Tables

**Table 1:** Characteristics of included studies.

Study ID	Study Design	Sample Size	Bethesda Classification	Molecular Test	Surgical Outcome
Carty SE et al., 2020 [22]	Retrospective cohort study	389 consecutive patients managed for 405 fine-needle aspirations (FNAs)	Bethesda IV	ThyroSeq v2 and v3 (multigene classifier)	Thyroidectomy for positive molecular test: 91% - Thyroidectomy for negative molecular test: 27%
Davis et al., 2020 [24]	Retrospective review	104 nodules in 100 patients	Atypia of Undetermined Significance (AUS) and Follicular Lesion of Undetermined Significance (FLUS) classified as Bethesda III. Follicular neoplasm/suspicious for follicular neoplasm	Afirma Gene Expression Classifier (GEC)	Resection: 14 (14%) of patients - Majority 86 (86%) did not undergo resection and were monitored clinically.
Fazeli SR et al., 2020 [26]	Retrospective cohort study	773 consecutive patients (393 Standard of Care, 380 Molecular test)	Bethesda III (AUS/FLUS): 69 cases (8.9% to 21.3%). Bethesda IV (FN/SFN): 24 cases (3.1% to 6.3%)	ThyroSeq v2	Overall rate of surgery: 23.4% in StC vs. 23.2% in molecular testing. Overtreatment rate decreased from 18.8% to 16.7%
Livhits et al., 2020 [32]	Randomized clinical trial	Total patients assessed: 2368 - Total nodules: 3140 - Indeterminate nodules: 427 - Included for analysis: 372 nodules from 346 patients	Bethesda III (AUS/FLUS): 316 (85%) - Bethesda IV (FN/SFN): 56 (15%) (After 55 nodules due to concurrent biopsy)	RNA test: Afirma genomic sequencing classifier DNA-RNA test: ThyroSeq V3 multigene genomic classifier	RNA test: - 12/107 benign results had surgery (all benign) - 58/73 suspicious results had surgery (31 malignant/NIFTP) DNA-RNA test: - 11/103 negative
Polavarapu et al., 2020 [37]	Retrospective analysis	376 patients with Bethesda III and IV nodules: - 262: No molecular testing - 50: GEC testing - 64: GSC testing	Bethesda III and IV indeterminate thyroid nodules only	Afirma Gene Expression Classifier (GEC) - Afirma Genomic Sequencing Classifier (GSC)	Surgical rates: - No molecular testing: 66.4% - GEC group: 60.0% (30/50) - GSC group: 46.9% (30/64)
San Martin VT et al., 2020 [40]	Retrospective analysis	299 nodules in 290 patients: - 178 nodules: GEC testing - 121 nodules: GSC testing	GEC group: - Bethesda III: 57.9% (103/178) - Bethesda IV: 42.1% (75/178)GSC group: - Bethesda III: 62.8% (76/121) - Bethesda IV: 37.1% (45/121)	Afirma Gene Expression Classifier (GEC)	GEC group: 47.8% (85/178) underwent surgery GSC group: 34.7% (42/121) underwent surgery
Song Y et al., 2020 [43]	Retrospective study	Total patients: 189 - Total nodules: 196 - Successfully tested: 188 nodules	Bethesda III: 153 nodules. Bethesda IV: 43 nodules	Next-generation sequencing (NGS) using FSZ-Thyroid NGS Panel V1	Of 84 surgically resected nodules: - 46 (54.8%) benign - 33 (39.3%) malignant - 4 (4.8%) intermediate
Sultan R et al., 2020 [45]	Retrospective analysis	98 patients with 101 nodules	Bethesda III and IV: 94 nodules - Nondiagnostic: 7 nodule	Afirma Gene Expression Classifier (GEC)	Of 32 GEC-suspicious nodules: - 18 underwent surgery - Surgical outcomes: - 7 benign (39%) - 1 FTC (6%) - 6 FVPTC (33%) - 4 NIFTP (22%)
Yang et al., 2020 [48]	Retrospective medical record review	27 cases total	Bethesda III: 21 cases - Bethesda IV: 6 cases	Multiplatform test (MPT): - ThyGenX/ThyGeNEXT - ThyraMIR	Positive MPT: 8/10 had surgery - Negative MPT: 2/15 had surgery
Abdelhakam et al., 2021 [19]	Retrospective cohort	133	Atypia or follicular lesion of undetermined significance (n = 65, 48.9%) - Suspicious for follicular neoplasm (n = 48, 36.1%) - Suspicious for Hürthle cell neoplasm	ThyroSeq (targeted next-generation sequencing)	Most patients (n = 87, 65.4%) did not undergo resection; decisions based on ThyroSeq results
Desai D et al., 2021 [25]	Retrospective analysis	415 cases (AUS/FLUS: 251; FN/SFN: 164)	Atypia of Undetermined Significance (AUS/FLUS) (Bethesda III): 251 cases - Follicular Neoplasm/Suspicious for Follicular Neoplasm (FN/SFN) (Bethesda IV): 164 cases	ThyroSeq V3 (genomic classifier)	127 cases underwent surgery: - 96 ThyroSeq positive cases - 31 ThyroSeq negative cases Histopathologic follow-up available for 127 cases.
Li, 2021 [31]	Retrospective review	202 participants	Bethesda III: 128 (63%) - Bethesda IV: 74 (37%)	ThyroSeq	Surgical resection performed in 49 out of 202 ITNs (24.3% overall). - Resection rates: 15.6% for Bethesda III and 39.2% for Bethesda IV
Lu, 2021 [33]	Retrospective evaluation of thyroid nodules that underwent molecular testing.	367 thyroid fine needle biopsies (FNBs) subjected to molecular testing, with 55 RAS-mutated cases identified.	Bethesda III (Atypical/FLUS) and IV (Follicular/Hurthle Neoplasm); specific numbers not detailed for all cases	ThyroSeq V2 and V3	40 patients underwent surgical resection based on cytology and molecular results. - 7 patients did not undergo surgery due to preference or comorbidities
Polavarapu P et al., 2021 [38]	Retrospective cohort analysis	468 indeterminate thyroid nodules: - 273: No molecular testing - 71: GEC testing - 124: GSC testing	Distribution: - Bethesda III (AUS/FLUS): 46% no testing, 60% GEC, 67% GSC - Bethesda IV FN: 42.1% no testing, 30% GEC, 17% GSC - Bethesda IV HCN: 12% no testing, 10% GEC, 16% GS	Afirma Gene Expression Classifier (GEC) - Afirma Genomic Sequencing Classifier (GSC)	Surgical rates: - No molecular testing: 68% - GEC group: (42/71) 59% - GSC group: (50/124) 40%
Selvaggi SM et al., 2021 [41]	Retrospective analysis	35 indeterminate thyroid nodules	Indeterminate nodules including FLUS - SFN/SHCN - SPTC	ThyroSeq V3	Pre-ThyroSeq: - 69% underwent surgery Post-ThyroSeq: - Positive results: 17/17 (100%) underwent surgery - Negative results: 4/18 (22%) underwent surgery
Zhu CY et al., 2021 [49]	Prospective follow-up of randomized controlled trial	Total nodules: 165 - Molecular testing: 158 patients with 165 nodules - Nonoperative management: 100 patients with 103 nodules	For nonoperatively managed nodules (n = 103): Bethesda III (AUS/FLUS): 97 (94.2%)Bethesda IV (FN/SFN): 6 (5.8%)	Afirma Gene Expression Classifier (GEC): 74 nodules - ThyroSeq v2: 91 nodules	Initial surgery: 73 nodules Delayed surgery: 12 nodules Nonoperative management: 100 patients with 103 nodule. Overall cancer/NIFTP prevalence: 19.4%
Akinsanya 2022 [20]	Retrospective cohort study	106	AUS/FLUS (Atypia of Undetermined Significance/Follicular Lesion of Undetermined Significance)	Afirma GSC (Gene Sequencing Classifier)	Surgery rates: - 67% (48/72) without Afirma testing - 44% (15/34) with Afirma testing; 73% of surgeries were in the Afirma-suspicious group.
Bao, 2022 [21]	Retrospective cohort study	41 adults with 43 indeterminate thyroid nodules	Indeterminate nodules classified as Bethesda III and IV (AUS/FLUS)	ThyroSeq V3 (multigene classifier)	Surgical resection rates: - Low-risk nodules: Only one underwent total thyroidectomy due to compressive symptoms
Dantey et al., 2022 [23]	Retrospective analysis	725 thyroid nodules biopsied during the study period	Non-diagnostic/unsatisfactory (B-I): 8% - Benign (B-II): 52% - Follicular Lesion of Undetermined Significance/Atypia of Undetermined Significance (B-III): 28% - Suspicious for Follicular Neoplasm/Suspicious for Neoplasm (B-IV): 6.2% - Suspicious for Malignancy (B-V): 2.3% - Malignant (B-VI): 3.2% - Specific B-III breakdown: FLUS (73%), AUS (27%	ThyGeNEXT-ThyraMIR (Interpace Diagnostics^®^)	One in four B-III category nodules (n = 52) underwent partial or total thyroidectomy; final pathology showed: Benign-32; Malignant-18; NIFTP-1.
Glass RE et al., 2022 [27]	Retrospective cohort study	648 indeterminate nodules total: - 305 underwent molecular testing - 343 no molecular testing	Bethesda III (AUS): 510 cases (78.7%) - Bethesda IV (SFN): 94 cases (14.5%) - Bethesda IV (SFN, Hurthle cell type): 44 cases (6.8%)	ThyGenX/ThyraMIR: 146 cases ThyroSeq V3: 136 cases	No molecular testing: 50.4% underwent surgery - ThyGenX/ThyraMIR: 30.8% underwent surgery - ThyroSeq V3: 38.2% underwent surgery
Kannan et al., 2022 [28]	Pilot study (observational)	19 patients underwent molecular testing	Bethesda II: 1 case - Bethesda III: 9 cases - Bethesda IV: 2 cases - Total Indeterminate Nodules: 11 cases	Thyro Track NGS panel covering 40 unique genes for Single Nucleotide Variations (SNVs) and Deletions (InDels), and 17 genes for known and unknown fusions (including BRAF, RAS, RET, NTRK, ALK)	Patients with positive NGS results advised hemithyroidectomy - Patients with negative or benign results advised sonographic follow-up.
Steinmetz et al., 2022 [44]	Retrospective analysis	142 indeterminate thyroid nodules	Bethesda III: 113 nodules (80%) - Bethesda IV: 29 nodules (20%)	ThyroSeq v2	73 nodules: underwent surgery - 69 nodules: avoided surgery - Management change in 91/142 (64%) nodules
Torrecilas ey al., 2022 [46]	Retrospective chart review	89 cytologically indeterminate nodules (CIN)	Bethesda III (AUS/FLUS): 49 (55%) - Bethesda IV (FN/SFN): 40 (45%)	ThyroSeq	38 nodules underwent surgeryMalignant: 15 (39%) - Benign: 23 (61%)
Olmos R et al., 2023 [35]	Single-center, prospective, noninterventional study	Total FNAs: 1272 Indeterminate nodules: 244 (19.2%) - Molecular testing performed: 155 cases (63.5%)	Distribution of indeterminate nodules (n = 244): - Bethesda III: 109 (8.6%) - Bethesda IV: 135 (10.6%)	ThyroidPrint^®^ (ten-gene classifier using qPCR) - Tests tumor microenvironment genes: CXCR3, CXCL10, CCR3, CCR7, CXADR - Tests epithelial cell genes: TIMP1, CLDN1, KTR19, AFAPL2, HMOX1	All 51 suspicious cases underwent surgery - Only 1/104 benign cases had surgery - Surgery rate decreased from 63% (non-tested) to 33% (tested)
Kim NE et al., 2023 [29]	Randomized controlled trial	369 indeterminate thyroid nodules	Bethesda III (AUS/FLUS): 314 nodules (85%) - Bethesda IV (FN/SFN): 55 nodules (15%)	Afirma Gene Expression Classifier (Afirma GEC) - Thyroseq v2 and Thyroseq V3 NGS panels	14/217 benign/negative results had immediate surgery - 113/133 suspicious/positive results had surgery - 15 nodules had delayed surgery
Papazian MR et al., 2023 [36]	Retrospective	96 patients with indeterminate thyroid nodules	Initial FNA results: - Bethesda III or IV (indeterminate) Repeat FNA results: - 55 (57%) remained indeterminate - 40 (42%) reclassified as benign - 1 (1%) reclassified as suspicious	ThyroSeq genomic classifier	ThyroSeq positive: 25/28 (89%) underwent surgery - ThyroSeq negative: 11/68 (16%) underwent surgery - Downgraded to Bethesda II: 37/40 (93%)
Lévesque F et al., 2024 [30]	Multicentric prospective study	500 consecutive patients	Bethesda III: 282 cases Bethesda IV: 218 cases	ThyroSeq V3 (TSv3)	TSv3 positive (n = 137): - 112 underwent surgery - 7 declined surgery - 18 outcome unknown TSv3 negative (n = 363): - 1 underwent surgery - 362 avoided
Munoz-Zuluaga CA et al., 2024 [34]	Retrospective study	otal FNAs: 1593 - Molecular testing collected: 624 cases - Cytologically indeterminate nodules: 148 - Successful Afirma GSC testing: 132 cases (89%)	Distribution of all FNAs (n = 1593) Bethesda I: 8.9% - Bethesda II: 65.4% - Bethesda III: 16.25% - Bethesda IV: 3.4% - Bethesda V: 1.25% - Bethesda VI: 4.8%	Afirma Genomic Sequencing Classifier (GSC) - Afirma Xpression Atlas (XA) as reflex test	8/11 XA-positive cases underwent surgery - 19/24 XA-negative cases underwent surgery. XA-positive (8 surgeries): - 7 papillary thyroid carcinoma
Raghunathan R et al., 2024 [39]	Subset analysis of a prospective randomized trial	136 patients with 149 Hürthle cell nodules: - 86 nodules: Afirma GSC - 63 nodules: ThyroSeq V3	GEC group: - Bethesda III: 57.9% (103/178) - Bethesda IV: 42.1% (75/178)GSC group: - Bethesda III: 62.8% (76/121) - Bethesda IV: 37.1% (45/121)	Afirma Gene Expression Classifier (GEC) - Afirma Genomic Sequencing Classifier (GSC)	GEC group: 47.8% (85/178) underwent surgery GSC group: 34.7% (42/121) underwent surgery
Sirotnikov S et al., 2024 [42]	Retrospective analysis	Total FNAs: 658 - ThyroSeq tested: 276 nodules	Bethesda III and IV (indeterminate)	ThyroSeq V3	74/116 positive cases underwent surgery - Surgical outcomes: - ROM with NIFTP: 77.5% - ROM without NIFTP: 68% - RAS-like ROM: 73.4%
Tumati A et al., 2024 [47]	Multi-institutional retrospective analysis	387 nodules from 375 patients - Successfully tested: 380 nodules	Bethesda III: 78.8% Bethesda IV: 21.2%	ThyGenX/ThyGeNEXT–ThyraMIR platform	Positive results: 74.4% underwent surgery - Negative results: 14.9% underwent surgery

**Table 2:** Summary of pooled mutation proportions by gene and platform.

Gene Mutation	Platform	Pooled Proportion (95% CI)	Heterogeneity
*BRAF*	ThyroSeq	2.7% (0.3%, 6.7%)	85.5%
*BRAF*	NGS	6.7% (3.4%, 10.7%)	0%
*RAS*	ThroSeq	18.8% (9.3%, 30.6%)	95.7%
*RAS*	NGS	15.7% (3.8%, 32.9%)	67.1%
